# Supplementary material for: Different patterns of foreground and background processing contribute to texture segregation in humans: an electrophysiological study
Source: PeerJ. 2023 Oct 2;11:e16139. doi: 10.7717/peerj.16139 (PMC10552746; doi:10.7717/peerj.16139)
Supplement: Supplemental Information 1 [file peerj-11-16139-s001.docx]

**Supplementary information for:**

**Different patterns of foreground and background processing contribute to texture segregation in humans: an electrophysiological study**

Baoqiang Zhang, Saisai Hu, Tingkang Zhang, Min Hai, Yongchun Wang, Ya li and Yonghui Wang

Corresponding author: Yonghui Wang

Email address: wyonghui@snnu.edu.cn

Corresponding author: Ya li

Email address: yali@snnu.edu.cn

**Supplemental Inventory:**

**Figure S1.** ERP waveforms and topography of formal experiment as a function of time (0–5,000 ms).

**Pilot psychophysics experiment 1.** The minimal luminance difference between the foreground and the background was determined using a 3 down 1 up staircase procedure.

**Figure S2.** Luminance difference threshold.

**Pilot experiment 2.** The aim was to examine whether the size of the texture stimulus affected TRF amplitude.

**Figure S3*.*** Stimuli of the pilot experiment 2.

**Figure S4.** Results of the pilot experiment 2.

**
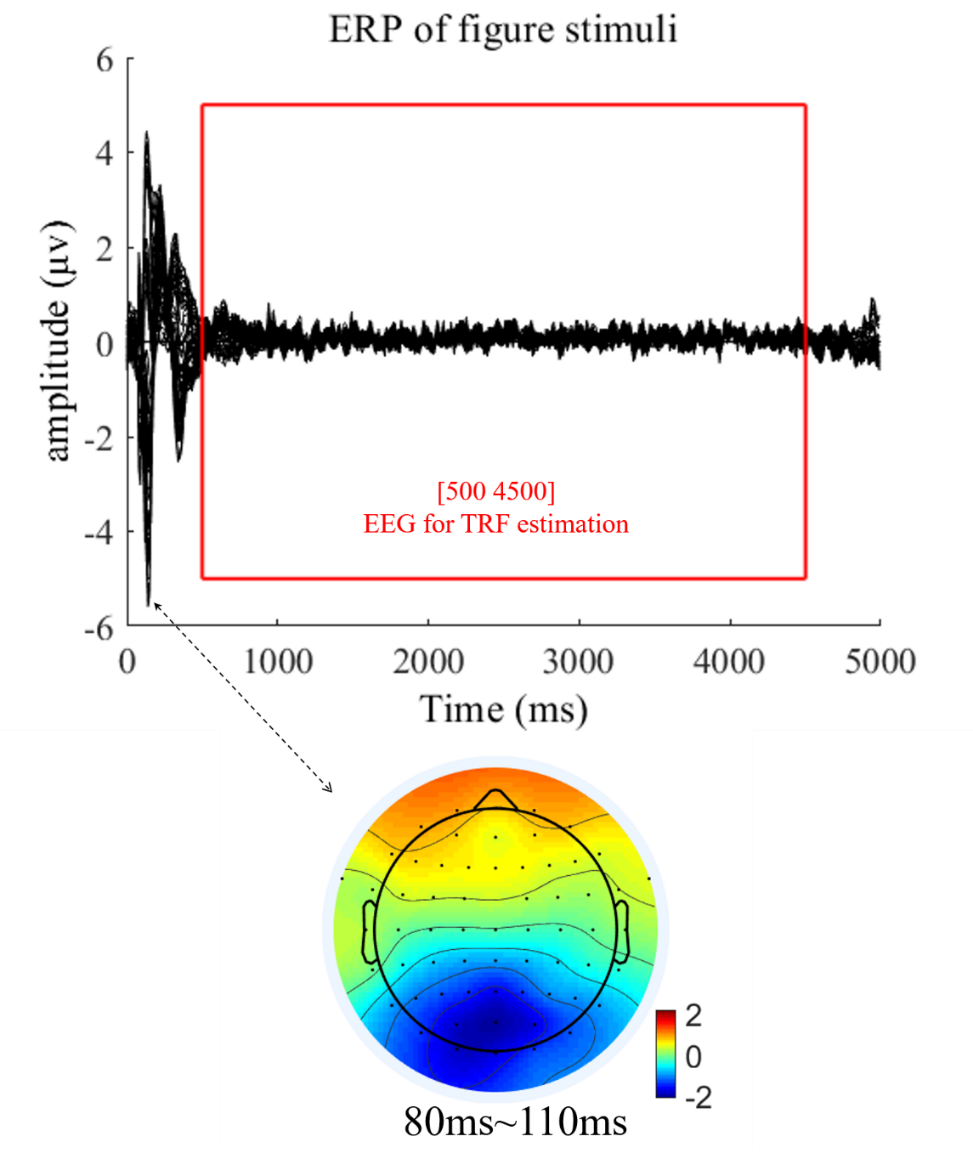
**

**Figure S1. ERP waveforms and topography of formal experiment as a function of time (0 – 5,000 ms).** Top: ERP waveforms of all channels for formal experiment as a function of time (0 – 5,000 ms). Bottom: Distribution map for the initial ERP onset response, mainly derived from Oz electrode. Note that to avoid the influence of the onset and offset response, which may bias the estimated temporal response function (TRF) results, we extracted the middle part (red rectangle) of the 5,000 ms EEG trial responses (500 – 4,500 ms) for further TRF calculation.

**Pilot psychophysics experiment 1**

The goal of this pilot psychophysics experiment was to measure the minimal luminance difference between the foreground and background part of the texture that is required to perceive the figure texture.

***Participants***

Fifteen subjects (2 males, aged 18-25 years) participated in this experiment. All participants had normal or corrected‐to‐normal vision and reported no history of neurological disorders. All participants provided written informed consent prior to the start of the experiment.

***Stimuli and task***

Figure texture used in the experiment were generated in the same way as figure texture of formal experiment. We set the luminance of foreground or background to maximum (100.94 cd/m^2^), and then adjusted the luminance of background or foreground to measure the minimal luminance difference between the foreground and the background to perceive the figure texture. Two staircase were mixed together: one (high to low staircase, HL staircase) where the initial difference between foreground and background luminance was set to 8.52 cd/m^2^ and all subjects were unable to complete texture segregation, and the other (low to high staircase, LH staircase) where the initial difference between foreground and background luminance was set to 57.90 cd/m^2^ and all subjects were able to complete texture segregation. This experiment includes six blocks, three of which are HL staircases and the other three are LH staircases. The participants were asked to judge whether the foreground was brighter in luminance than the background... This experimental apparatus is the same as the formal experiment.

***Results***

The luminance difference threshold between foreground and background was 21.43 ± 3.64 (cd/m^2^). The luminance difference thresholds of all subjects were showed in Figure S2. To ensure 99% of subjects could distinguish between foreground and background, the minimal luminance difference between the foreground was set to 29.91 cd/m^2^ (*M* + *SD**2.33).


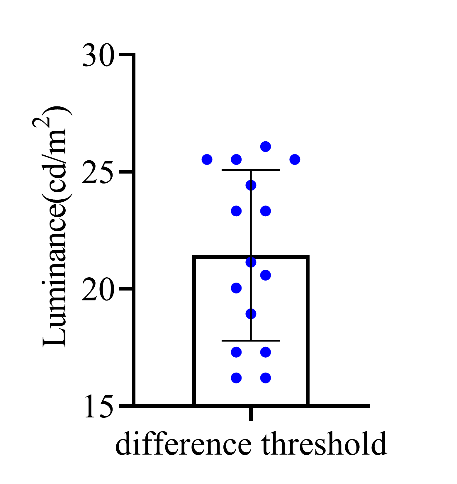


**Figure S2.** **Luminance difference threshold.** The difference threshold of each subject are presented in the figure. Error bars denote 1 SD calculated across subjects and blue dots denote the data from each subject.

**Pilot experiment 2**

The goal of this experiment was to check whether the size of the texture stimulus affected TRF amplitude.

***Participants***

Eight adult participants (1 male, aged 18-23 years) were recruited for this experiment. All participants had normal or corrected‐to‐normal vision and reported no history of neurological disorders. All participants provided written informed consent prior to the start of the experiment.

***Stimuli***

The materials and apparatus were the same as the formal experiment, except that only small (8°×8°) and large uniform (34°×25°) textures were included in this experiment, see in the Figure S3.


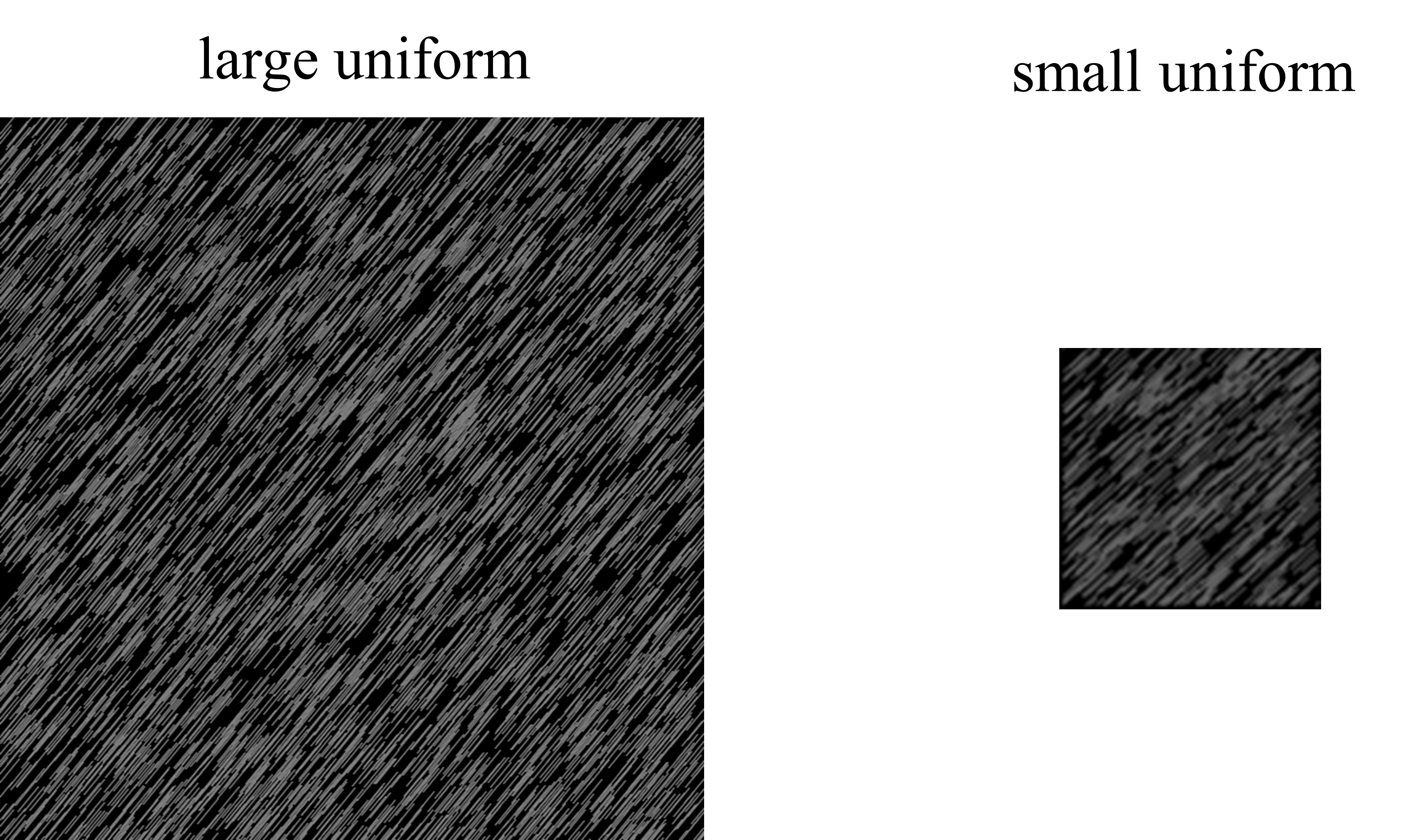


**Figure S3*.* Stimuli of the pilot experiment 2**. Luminance-defined figure and uniform texture are presented in the central visual field (Left: large uniform texture; Right: small uniform texture).

***Experimental procedure***

Experimental procedure is the same as the formal experiment.

***EEG data acquisition and preprocessing***

EEG data acquisition and preprocessing is the same as the formal experiment. We have upload the raw data to the Figshare (*http://dx.doi.org/10.6084/m9.figshare.22434547*)

***TRF calculation***

TRF calculation is the same as the formal experiment. The TRF responses for large uniform were extracted from the EEG recordings in large uniform textures, and those for small uniform were calculated from the EEG data in small uniform textures, based on the corresponding luminance temporal sequences.

***Statistical analysis***

After visual inspection, the mean amplitude of N1 (70-110 ms), P1 (120-150 ms) and N2 (160-230 ms) components, were calculated. Next, we examined whether each component was different between brain regions and uniform conditions. Repeated-measures ANOVAs with uniform conditions (large and small uniform) and topographical factors (occipital, parieto-occipital, parietal, central, frontal) were performed separately for each component.

***Results***

**
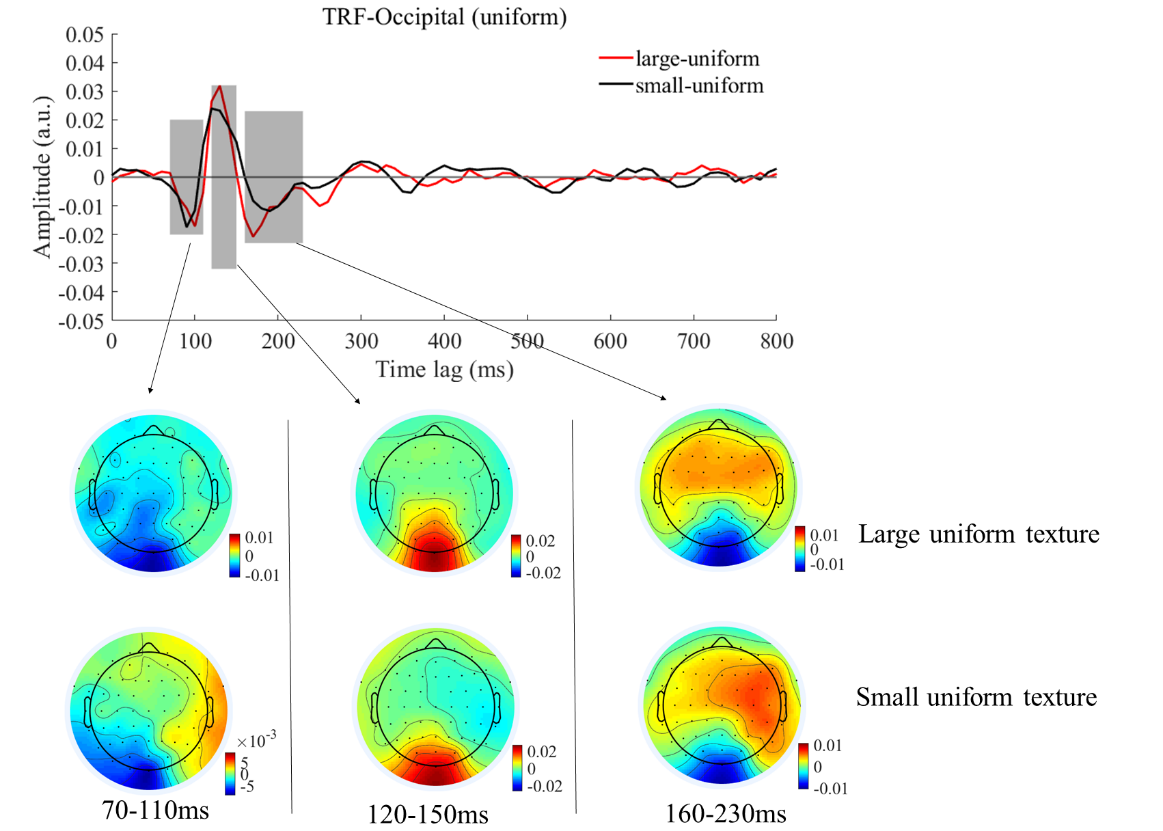
**

**Figure S4. Results of the pilot experiment 2.** Top**:** Original TRF waveforms of the large and small uniforms in the occipital region as a function of temporal lag (0 to 800 ms). Bottom: Topographies of the three components (70-110 ms, 120-150 ms and 160-230 ms) of the large and small uniforms.

*N1 component at 70-110 ms*

To test whether the size of uniform affects TRF amplitude, we performed a 2 (uniform conditions: large, small) ×5 (topographical factors: occipital, parieto-occipital, parietal, central, frontal) repeated measures ANOVA. Both the main effect of uniform conditions (*F*_(1,7)_ = 0.500, *p =* 0.502, $\eta_{p}^{2}$ = 0.067), the main effect of topographical factors(*F*_(4,28)_ = 0.336, *p =* 0.594, $\eta_{p}^{2}$ = 0.046) and the interaction between stimulus conditions and topographical factors (*F*_(4,28)_ = 0.035, *p =* 0.940, $\eta_{p}^{2}$ =0.005) were not significant.

*P1 component at 120-150 ms*

To statistically test whether the different TRF patterns exist between large and small uniform conditions, two-way repeated measures ANOVA was performed with 2 (uniform conditions: large, small) ×5 (topographical factors: occipital, parieto-occipital, parietal, central, frontal). The main effect of topographical factors was significant (*F*_(4,28)_ = 18.976, *p <*0.001, $\eta_{p}^{2}$ = 0.731), whereas neither the main effect of uniform conditions (*F*(1,7) = 0.785, *p* = 0.405, $\eta_{p}^{2}$ = 0.101) nor the interaction between these factors (*F*(4,28) = 0.200, *p* = 0.752, $\eta_{p}^{2}$= 0.028) was significant.

*N2 component at 160-230 ms*

To statistically confirm the different TRF patterns between large and small uniform conditions was exist, two-way repeated measures ANOVA was conducted with 2 (uniform conditions: large, small) ×5 (topographical factors: occipital, parieto-occipital, parietal, central, frontal). The main effect of topographical factors were significant (*F*(4,28) = 11.228, *p* = 0.008, $\eta_{p}^{2}$ = 0.616). The main effect of uniform conditions was not significant (*F*(1,7) = 0.397, *p* = 0.548, $\eta_{p}^{2}$ = 0.054). The interaction between uniform conditions and topographical factors were not significant (*F*(4,28) = 4.418, *p* = 0.063, $\eta_{p}^{2}$ = 0.187).

These results indicate that the size of the texture stimulus not affected TRF amplitude.
